# The association between internet addiction and sleep quality among medical students in Saudi Arabia

**DOI:** 10.1080/07853890.2024.2307502

**Published:** 2024-01-31

**Authors:** Mohammad Ahmed Hammad, Mohammed Hussain Feheed Alyami, Huda Shaaban Awed

**Affiliations:** College of Education, Najran University, Kingdom of Saudi Arabia

**Keywords:** Internet addiction, sleep quality, Medical students, Saudi Society

## Abstract

**Background:**

Sleep is one of the fundamental human needs, essential for maintaining a high quality of life and mental and physical well-being across all age groups. Poor sleep quality often stems from negative lifestyle habits, including excessive internet usage. Therefore, it is important to determine the prevalence of internet gaming disorder among youth in Saudi Arabia and to examine the relationship between internet addiction levels and sleep quality.

**Methods:**

Data were collected from 338 medical students in the southern region of Saudi Arabia (mean age = 21.2 years, standard deviation = 3.29 years). Participants completed an online questionnaire comprising the Internet Addiction Test (IAT) and the Pittsburgh Sleep Quality Index (PSQI). Data analysis employed iterations, chi-square tests, Pearson correlation coefficients, and ANOVA.

**Results:**

The results revealed that 21% of the participants exhibited severe internet addiction, while 31% displayed moderate internet addiction. Furthermore, the findings indicated a positive correlation between sleep quality and the severity of internet addiction. Sleep quality symptoms explained 75% of the variance in Internet addiction scores, even after controlling for demographic variables. Additional bivariate analyses revealed that individuals who spent six or more hours online daily were more likely to experience symptoms of poor sleep quality and exhibit a higher severity of internet addiction. Additionally, Men were more susceptible to developing internet addiction compared to women. Moreover, students with internet addiction tended to have lower academic achievements.

**Conclusion:**

These findings, while exploratory, offer valuable insights into potential interventions, strategies, and programs for mitigating internet addiction and enhancing sleep quality among medical college students.

## Introduction

The rapid expansion of the internet has brought numerous benefits to our lives. However, excessive internet usage has also given rise to various personal and societal issues [1]. Among these negative behaviours, one of the most significant is internet addiction [2]. The concept of internet addiction was initially formulated by Goldberg [3]. Subsequently, the term "pathological internet" emerged, along with various alternatives like compulsive internet use and problematic internet [4]. Nevertheless, "internet addiction" remains the most commonly used term. Due to the increasing concern regarding internet addiction among parents, specialists, and officials, numerous recent studies have been conducted, encompassing all age groups, including children and adults [2,5–9]. Many of these studies have confirmed that internet addiction leads to various physical problems, such as sleep disorders [8], depression, anxiety [10], Alcohol abuse [11], aggressiveness [12], and disruptions in circadian rhythms [13]. Additionally, it is associated with personal issues like poor academic achievement [14], and social isolation, as well as work-related problems [15]. Moreover, internet addiction has economic repercussions, resulting in financial losses [16]. Furthermore, it has led to a disturbance in daily activities, especially among university students and medical college students, resulting in negligence of assignments and coursework [17].

Sleep plays a pivotal role in human life, influencing both physical and psychological health. Adequate sleep is crucial for the secretion of growth hormones, vital for normal physical development [18]. Consequently, poor sleep quality leads to numerous adverse consequences [19], such as headaches, learning difficulties, memory impairment, aggressive behaviours, and diminished cognitive processes, including memory and learning abilities. These issues jeopardize the psychological, physical, and academic well-being of students [20,21]. Poor sleep quality is also linked to mental disorders and chronic diseases, such as cardiovascular diseases and diabetes [22]. Several studies have established a connection between poor sleep quality and excessive internet use [8,23,24]. Internet addiction adversely affects circadian rhythms, leading to insomnia and other sleep disorders. The use of electronic devices in bed disrupts sleep due to cognitive, emotional, or physiological stimuli [13]. Exposure to bright light from electronic devices causes alterations in sleep onset. Particularly, short-wavelength emissions from electronic devices delay the onset and phases of sleep [23]. Additionally, excessive internet usage may result in gray matter atrophy, negatively impacting an individual’s concentration and decision-making abilities [25]. Poor sleep quality further impairs cognitive processes like memory and learning abilities, putting students’ academic performance at risk and leading to physical and psychological health problems, as well as disruptions in other growth and development processes [20]. However, the increasing prevalence of Internet use *via* various means, including computers and smartphones, has made it a habitual practice for students to use the Internet before bedtime [13,26,27]. Several research studies have assessed the sleep quality of individuals diagnosed with internet addiction. For example, one study [28] found that students who spent substantial time on social networking sites and watching TV were more likely to experience sleep problems and depressive symptoms. A study conducted in Korea with middle school students revealed a significant association between reduced sleep time and excessive internet use [29]. Another study reported [30], that internet addiction, particularly internet gaming, was inversely correlated with quality of life in both subjective (life satisfaction) and objective (Environmental Quality) indicators. Furthermore, a study identified that adolescents or young adults experiencing difficulty falling asleep or maintaining sleep were more prone to becoming addicted to the Internet. Individuals heavily dependent on the Internet al.so had disruptions in their basic daily rhythms [8].

### Current study

Saudi Arabia ranks 17th globally in terms of internet penetration rate, with a population of 34.218 million, Out of this population, 32.23 million people are internet users, with 25 million using social networks, averaging two hours and fifty minutes of daily use [31]. The Pew Research Centre reports that smartphone ownership among individuals in Saudi Arabia ranges from 60% to 95%, and this percentage continues to rise [32]. Nearly 99% of adults in Saudi Arabia own smartphones [25]. The internet encompasses various social media and smart applications, extensively used in education, healthcare, and entertainment. In education, smartphones enable students to search for information easily and access content related to their subjects [33]. In the healthcare sector, numerous smartphone applications aid medical students, patients, and professionals in medical training and education [33,34]. Saudi Arabia hosts approximately 36 universities with over 1.7 million students [8]. Despite limited studies in Saudi Arabia that have examined the prevalence of internet addiction and its correlation with certain variables [5,8,25,29,33,35,36]. There is a dearth of research on internet addiction and sleep quality among university students, particularly those in medical specialties. Addressing this gap in the literature, our study aims to determine the prevalence of internet addiction among medical college students and explore its relationship with sleep quality. We seek to investigate whether internet addiction predicts sleep quality in medical college students through a population-based cross-sectional study. Additionally, we aim to discern how the level of internet addiction varies based on demographic variables such as gender, GPA (academic performance), time spent on recreational activities, place of residence, and smoking habits.

## Methodology

### Participants

This cross-sectional exploratory study utilized a simple random of undergraduate students of a medical colleges from universities (Najran, King Khalid, Jazan) in the southern region of the Kingdom of Saudi Arabia. The required sample size was determined using G*Power 3.1 [37]. Introducing an error probability of 0.05 (α) and a power of 0.90, the recommended sample size for ANOVA analysis with multiple predictions was 332. Therefore, to account for response rate adjustments, invitations to participate in the study were sent to 374 students. After excluding disapprovals and incomplete responses, the final sample consisted of 338 students, which exceeded the recommended sample size for this study. To be eligible for the study, students needed to be over 18 years of age, currently enrolled in a 4-year degree program at their university during the 2022–2023 academic year, proficient in Arabic or English, and willing to participate. Table 1 presents the characteristics of the final sample.

**Table 1.** Demographic characteristics of the study participants.

Demographic Characteristics		*N*	*Percentage*
Age		–	21.30 (4.96) years
Sex	Men	178	52.66%
	Women	160	47.33%
living place	With family	224	66.27%
	University Housing	114	33.72%
smoking	Non smoker	242	71.59%
	Smoker	96	28.40%
Time spends on the Internet every day	≤3 h	50	14.8%
	4–5 h	162	47.9%
	≥6 h	126	37.3%
GPA (performance)	≤ 3.9	111	32.8%
	4-4.4	147	43.5%
	> 4.5	80	23.7%

### Data collection

#### Procedure

Data were collected through an online survey from April to May 2023. Invitations to participate in the study were sent to randomly selected potential participants from medical colleges at Najran, Jazan, and King Khalid universities in the southern region of the Kingdom of Saudi Arabia. The invitation included an information package and a study questionnaire using Google Forms. The information package explained the study’s purpose and procedures, as well as the rights of participants (including anonymity, confidentiality, voluntary consent, non-victimization, and the freedom to withdraw consent at any time during the study [38,39]. Only those who provided informed consent after reviewing the package proceeded to complete the questionnaire. The study and its procedures were approved by the Deanship of Scientific Research at Najran University (NU/IFC/2/SEHRC/-/19).

#### Socio-demographic questionnaire

The questionnaire included questions about participants’ gender, living arrangements (with family or in university housing), smoking habits, daily internet usage, and GPA (academic performance).

### Internet addiction test (IAT)

The IAT test, developed by [40], was employed [41.] adapted this test to the Arabic language. The test comprises 20 items designed to measure the level of internet addiction. It employs a six-point Likert scale with values ranging from 0 to 5 (0 = Never; 1 = Rarely; 2 = Sometimes; 3 = Frequently; 4 = Often; 5 = Always). Total scores range from 0 to 100 points. Participants are categorized into several groups based on their scores [42]. 0 to 19 indicates the absence of internet addiction, 20 to 39 indicates a low level of internet addiction, 40 to 69 represents an average level of internet addiction, and 70 to 100 indicates a severe level of internet addiction. The Arabic version of IAT has demonstrated good reliability and validity (Cronbach’s alpha = .92) [41]. In this study, Cronbach’s coefficient α for IAT was 0.87.

### The pittsburgh sleep quality index (PSQI)

The PSQI scale developed by [43] was utilized and adapted to Arabic by [44]. PSQI comprises 19 items designed to assess sleep quality and its disorders over the past month in clinical groups. The 19 items are categorized into 7 components, including (1) Sleep Duration, (2) Sleep Disturbance, (3) Sleep Latency, (4) Daytime Dysfunction due to Drowsiness, (5) Sleep Efficiency, (6) Overall Sleep Quality, and (7) Use of Sleep Medications. Each component yields a score ranging from 0 to 3, with 3 indicating the highest dysfunction. The scores from the seven components are summed to obtain a single total score, ranging from 0 to 21, with higher scores indicating poorer sleep quality. A cut-off score of ≥ 5 was chosen to distinguish between individuals who sleep poorly (≥5) and those who sleep well (<5; 43]. The Arabic version of PSQI has shown good reliability and validity (Cronbach’s alpha = .65) [44]. In this study, Cronbach’s coefficient α for PSQI was 0.72.

### Data analysis

Following data collection, all questionnaires were scrutinized for completeness, accuracy, and internal consistency. Statistical analyses were conducted using SPSS version 21. Descriptive statistics (means and standard deviations or frequencies and percentages) were used to describe categorical variables and continuous variable means. Bivariate and multivariate comparisons employed appropriate statistical tests such as t-tests, chi-square tests, and ANOVA, following checks for normality and homogeneity. Correlation and logistic regression analyses were also conducted to examine the association between internet addiction and sleep quality while controlling for relevant demographic variables. A significance level of *p* ≤ 0.05 was set for all statistical tests. statistical tests performed and effect size estimates were calculated and classified (0.2 was considered small, 0.5 as medium, and 0.8 as large) using guidelines from Cohen [45].

## Results

### Prevalence of internet addiction

Table 2 presents the prevalence of internet addiction among the current sample of medical college students in the southern region of the Kingdom of Saudi Arabia. The classification is based on [42], categorizing addiction levels as absent, low, medium, and severe. The distribution of the sample across these levels is as follows: approximately 21.0% exhibited severe internet addiction, followed by nearly 31.1% with moderate internet addiction, 37.0% with low internet addiction, and about 10.9% showed no signs of internet addiction.

**Table 2.** Prevalence of internet addiction among medical college students.

Internet Addiction scores	Internet addiction level	Frequency (*N* = 305)	Percentage
≤19	No internet addiction		10.9%
20 to 39	Low internet addiction	125	37.0%
40 to 69	Moderate internet addiction	105	31.1%
≥70	Severe internet addiction	71	21.0%

Table 3 presents the results of a monovariance analysis conducted to examine differences in the level of internet addiction based on gender/place of living, smoking, time spent by a student online, GPA (performance). The results indicated that there were significant differences at the level of *p* < 0.01 for all variables, it is clear that the levels of internet addiction in the study sample vary based on these demographic characteristics.

**Table 3.** *ANOVA* results examining internet addiction scale scores by Sex, living place, time spends on internet every day, smoking, and GPA among medical college students.

Source	*Type III Sum of Squares*	*df*	*Mean Square*	*F*	*Sig.*	*Effect size*	*Level of effect size*
GPA (performance)	7963.073	2	3981.573	21.077	.000	.71	Medium
Sex	951.511	1	951.511	5.037	026	.58	Medium
living place	1970.727	1	1970.727	10.432	.001	.63	Medium
Time spends on internet every day	2891.656	2	1445.828	7.654	.001	.60	Medium
smoking	1551.074	1	1551.047	8.211	.004	.58	Medium
Error	52704.825	279	188.906				
Total	842996.000	338					

^a^ *R^2^* = .770 (*Adjusted R^2^* = .720.

To further assess these differences, the Scheffe test was employed to compare internet addiction levels based on students’ daily internet usage and GPA (performance) (see Table 4).

**Table 4.** Scheffe test Comparison of differences in internet addiction based on time spent on the internet every day and GPA (performance).

Variables	(J) Number of children	Mean *Difference (I-J)*	*Std. Error*	*Sig.*	95% Confidence Interval	
					Lower Bound	Upper Bound
Time spends on the Internet every day						
≤3 h	4–5 h	−9.4477*	2.2235	.00	−14.9197	−3.9756
	≥6 h	−27.6425*	2.2972	.00	−33.2960	−21.9891
4–5 h	≤3 h	9.4477*	2.2235	.00	3.9756	14.9197
	≥6 h	−18.1949*	1.6325	.00	−22.2126	−14.1772
≥6 h	≤3 h	27.6425*	2.2972	.00	21.9891	33.2960
	4–5 h	18.1949*	1.6325	.00	14.1772	22.2126
GPA (performance)						
≤ 3.9	4-4.4	−10.1541*	1.7282	.00	−14.4073	−5.9009
	> 4.5	−30.8764*	2.01573	.00	−35.8370	−25.9157
4-4.4	≤ 3.9	10.1541*	1.72827	.00	5.9009	14.4073
	> 4.5	−20.7223*	1.90956	.00	−25.4216	−16.0230
> 4.5	≤ 3.9	−30.8764*	2.01573	.00	25.9157	35.8370
	4-4.4	−20.7223*	1.90956	.00	16.0230	25.4216

* The mean difference is significant at the *.05* level.

Table 4 confirms statistically significant differences in internet addiction levels among the study sample based on both daily internet usage and GPA. Specifically, students who used the internet for ≤3 h had significantly lower internet addiction scores compared to those who used it for 4-5 h or more than 6 h. Additionally, students with a GPA of ≤3.9 exhibited a higher level of internet addiction than those with a GPA of 4-4.4 or greater than 4.5, suggesting a negative impact of internet addiction on academic performance.

Table 5 shows the results of the *t*-test conducted to compare the scores of internet addiction based on the living space and smoking of the participants. The results revealed that internet addiction was more pronounced in men students (*M* = 51.48, SD = 19.36). Compared to their women peers (*M* = 38.57, SD = 18.41). The results also indicated that those who do not live with their families (*M* = 55.89, SD = 19.19), compared to students who live with their families (*M* = 40.01, SD = 19.69). The results also indicated that students who smoked had a higher level of internet addiction (*M* = 56.68, SD = 19.05). Compared to students who do not smoke (*M* = 40.88, SD = 19.51). Hence, the results suggest that students who do not live with their families, and students who smoke may have a high level of internet addiction compared to their peers who live with their families or who do not smoke.

**Table 5.** *t-test* analysis of internet addiction based on Sex, living place, and smoking.

Variables	*N*	*M*	*SD*	*t*
Sex				
Men	178	51.48	19.36	5.95*
Women	160	38.57	18.41	
living place				
With family	224	40.01	19.69	7.06*
University Housing	114	55.89	19.19	
Smoking				
Non smoker	242	40.88	19.51	6.58*
Smoker	96	56.68	19.05	

* Statistically significant at *p < 0.05* level.

### Internet addiction and sleep quality

The average scores on the PSQI sleep quality scale for the sample (*M* = 7.84, *SD* 3.22), with scores ranging from 0 to 21. ANOVA Test was conducted to examine the differences in sleep quality scores between the internet addiction groups and showed that those in the severe Internet Addiction Group received significantly higher scores on the measures of sleep quality (PSQI) than the rest of the internet addiction groups. It also turns out that most of the sample had poor sleep quality according to the cut-off score ≥5, as the average scores of the moderate and severe Internet Addiction Group on the PSQI scale were above 5 degrees, Table 6.

**Table 6.** *ANOVA* test Comparison of internet addiction groups on sleep quality (*N* = 338).

	Group	*f*	*M*	*SD*	*F*	*p*	*Post Hoc*
							*Scheffe*
(PSQI)	No internet addiction	37	4.13	0.91	239.331	.000	(D) > (C) > (B) > (A)
	Low internet addiction	125	4.98	1.81			
	Moderate internet addiction	105	9.38	2.14			
	Severe internet addiction	71	11.53	1.65			

*Note*: (A) No internet addiction; (B) Low internet addiction; (C) Moderate internet addiction; (D) Severe.

Furthermore, Pearson’s correlation analysis indicated a fairly strong positive correlation (*r* = .83, *p < .001*) between internet addiction scores and sleep quality scores, signifying that as internet addiction scores increased, sleep quality scores also increased, and vice versa.

Finally, a linear regression analysis was conducted to assess the relationship between internet addictions and sleep quality (see Table 7). Controlling for five demographic variables, sleep quality was found to predict 75% of the variation in scores (*R* = 0.869; *R^2^* = .755; *Adjusted R^2^* = 0.750; *F* = 67.32, *p < .05*).

**Table 7.** Linear regression analysis of the association between internet addiction and sleep quality.

	*Unstandardized coefficient*		*Standardized coefficient* *Beta*	*t*		*P*
Variables	*B*	*Std. Error*			f	
Sleep Quality	0.638	0.225	0.638	18.41	67.32	0.00

Note. Sex, living place, Time spends on the internet every day, smoking, and GPA were included as control variables.

## Discussion

This survey, to our knowledge, is the first of its kind to investigate the relationship between internet addiction and sleep quality among students in medical colleges at universities in southern Saudi Arabia. The study revealed that approximately 21% of the participants exhibited severe internet addiction, with around 31% demonstrating moderate internet addiction. This suggests that nearly half of the sample displayed moderate to severe addiction, which aligns with findings from several previous studies [46–49]. However, these results also differ from some studies, where prevalence was relatively lower, such as 18.3% in the UK, 6.6% in Italy [50], and 8.2% in Greece [51]. These variations could be attributed to cultural factors, differences in diagnostic criteria, assessment questionnaires, and the selective samples used in online surveys.

It should be noted that most of the studies reporting lower incidence rates (8–18%) were conducted before the COVID-19 pandemic. The high rates of internet addiction observed in this study may be attributed to the pandemic-induced lockdowns, forcing students to rely extensively on the internet for learning. The effects of the pandemic and associated social isolation have been linked to increased internet and gaming addictive behaviour among students in various countries. This surge in internet addiction cases can be attributed to increased stressors during the pandemic, limited access to mental health services, and the substitution of online activities for traditional in-person ones. Despite the varying reasons for these results, the alarming prevalence of severe internet addiction (more than 21%) and moderate internet addiction (31%) in this study underscores the need for further research to comprehensively understand the risk and protective factors associated with internet addiction in young people.

The current study also found that sex, smoking, place of living, time spent online, and GPA level were important factors influencing the level of internet addiction. The high prevalence of internet access and ease of access to it from Saudi Arabia, whether through smartphones or tablet computers, may be one of the factors contributing to the excessive use of the internet. Some social media applications (such as WhatsApp, Snapchat, and Twitter) are also particularly popular [31]. Inclement weather in Saudi Arabia (for example, extreme heat during the summer months) is likely to increase their use of digital media by forcing them to stay indoors during the day [49]. In this study, men medical college students scored higher on internet addiction compared to women. The high level of internet addiction in men students can be explained by the fact that they spend much more time using the internet and playing games than women students. This is consistent with similar results in other studies [52,53], in which individuals suffering from internet addiction tend to be men at a young age. Our results also indicated that about 47.9% spend 4–5 h a day online, while 37.3% of the sample spend 6 h or more. This excessive use of the internet through the use of the Internet of 4–5 h or 6 h or more per day will lead to a state of internet addiction. Internet addiction was found to be significantly higher among those who used the internet for 6 h or more. These results were not surprising given that many previous studies have linked the duration of internet use to a higher risk of internet addiction. For example, a study [54] found that 40.8% use the internet for 5-7 h a day, and have a severe level of internet addiction. As indicated by the study [46] that those with a high level of internet addiction use the internet at a rate of more than 4 h a day, as reported by another study that more than a third of the 39.4 sample use the internet more than 5 h a day, which made them have a high level of internet addiction [55]. Hence, the current results of previous studies and analyses suggest that we need to keep in mind the amount of time young people spend using the internet because it is a strong risk factor for the development of internet addiction. In the same context, internet addiction may arise as a result of not adhering to good study habits. Where the internet may act as a substitute for study habits, it may cause a significant drop in grades, absenteeism from classes, and poor consolidation in sub-achievements [56]. As well as excessive use of the internet, it may in turn lead to a lack of sleep, which leads to poor effort and the inability of students to perform the assignments that are asked of them. This is indicated by the results of our studies that academic performance, measured by self-reported GPA, is significantly lower in students addicted to the Internet. This is also confirmed by numerous studies, for example, the results of a study [57] that almost two-thirds of students have had their academic performance affected by excessive internet use, and one-third of students have also stated that their classroom attendance has been affected by excessive internet use. Also, a study [54], that the degree of internet addiction was higher among those with a lower GPA. In the same context, a study by [53,58] that internet addiction was significantly negatively related to the academic performance of undergraduates.

The results indicated that participants who smoked had higher scores in Internet Addiction compared to those who did not smoke, the literature links internet addiction with smoking, and some studies have suggested that smokers may more easily develop internet addiction [59]. Furthermore, a study of 467 Chinese adolescents showed that individuals with internet addiction, and internet gaming disorder may engage in more risky behaviours such as smoking, truancy, drinking, and fighting [60]. In addition, internet activity, gaming cues, and nicotine all alter the brain’s neural networks, which may have a long-term impact on cognitive functions such as memory and sleep quality [61]. It was pointed out by [62] that students who smoke change their sleep patterns and are less likely to have good sleep quality. As indicated by the results of the study [63,64] people who reported poor sleep quality also smoked excessively and indulged in frequent internet surfing. Also, the results indicated that internet addiction is increasing among students who live in university housing or away from their families, and this seems logical in Saudi society where the family plays a crucial role in the development of internet addiction. Young Saudis are subject to high levels of parental control, and therefore those who live with their families are subject to this parental control, so they use the internet less, in addition to carrying out many responsibilities and household tasks besides their studies. This is confirmed by [65], who that parents are more careful to follow their children, especially when they use the internet.

Referring to the main focus of this study, which is the correlation between internet addiction and sleep quality, the results of the ANOVA test and the gradual hierarchical regression together (Tables 5, 6) confirmed that higher internet addiction scores were associated with lower sleep quality. More than half (52%) of students with moderate to severe internet addiction reported poor sleep quality. Sleep quality has been linked to internet addiction, and 75% of the variation in Internet addiction scores was explained after controlling for the effects of sex, smoking, place of living, time spent online by the student, and GPA level. These results support the assumption that internet addiction has a negative impact on sleep quality and is a risk factor for the deterioration of sleep quality, which is mainly characterized by insufficient sleep, poor sleep quality, and symptoms of daytime sleepiness [8,64]. There may be several explanations that try to clarify this connection. For example [66], suggested that using the internet at night may directly replace bedtime. This was also confirmed by [67] that poor sleep quality is closely related to everyday life behaviours including excessive internet use. Excessive use of the internet and the online behaviours involved (such as online gaming) can lead to sleep problems such as sleep deprivation [66]. Online games and watching action movies stimulate the central nervous system, which contributes to prolonged sleep delays [64]. In addition [63] , noted that internet addiction contributes to the disruption of the circadian rhythm, which may adversely affect sleep time and sleep duration, leading to daytime fatigue and poor work performance. Another possible explanation is the emission of blue light through screens known to suppress the secretion of melatonin by the pineal gland, which leads to a prolongation of sleep time [66]. In addition, poor sleep, especially among medical college students, is harmful and has many other disadvantages, for example, it may increase the likelihood of medical errors during practical training in college.

Together, the current findings add to the existing literature on internet addiction, especially among young people. However, the study has several limitations that must be taken into account when interpreting and generalizing these results. Firstly, it is difficult to generalize the results to other population groups because this study was conducted on a small sample of students of health faculties at universities in southern Saudi Arabia. Also, we admit that we were only able to clarify the relationship between internet addiction and sleep quality, but not to determine the causal relationship due to the methodology of our research, our research study is only exploratory and aims to highlight the relationship between internet addiction and sleep quality. Moreover, the use of an online survey with self-administered questionnaires and self-reports on the duration of online games leads to a bias in the methodology, the data set, and the results derived from it. Finally, future studies need to separate the individual and collective impact of these factors on internet addiction and sleep quality among college students. Most of the above methodological limitations can be addressed by conducting prospective studies on larger and more diverse samples, and by longitudinal and qualitative studies to determine the causal relationship and clarify the underlying mechanisms of influence.

## Conclusions and implications

The primary objective of this study was to investigate the relationship between internet addiction and sleep quality among students enrolled in medical colleges at universities in the southern region of Saudi Arabia (Najran, Jazan, King Khalid). Despite its limitations, this study contributes to our understanding of internet addiction among medical college students. In particular, it strengthens the argument that internet addiction, often viewed as distinct from substance dependence, shares characteristics and mechanisms similar to substance use disorders. This insight carries significant implications for interventions and public health policies. Of particular concern, and in line with existing literature, is the association between addictive behaviours like internet addiction and various psychological disorders, including sleep disorders [8,24].

Young people constitute the future of any society. As university students, they are expected to make substantial contributions to the labour market, their families, and society as a whole. However, internet addiction and mental disorders not only hinder their potential but also incur substantial costs, including school dropouts, job loss, medical expenses, school or work absenteeism, long-term disabilities, and, ultimately, the risk of premature death due to suicide or stress-related lifestyle disorders. Consequently, the current findings underscore the necessity of developing interventions and strategies to prevent internet addiction, particularly among adolescents and young adults.

One approach is the implementation of diverse and engaging early education curricula designed to build resilience against internet addiction in adolescents and young adults. Parents should also pay special attention to the amount of time their children spend on the internet and the frequency of their online gaming activities. Moreover, efforts should be directed towards strengthening psycho-emotional and social support systems for young people, involving parents, teachers, and social psychologists in providing timely support and addressing their psychosocial needs. Preventive measures and early interventions play a pivotal role in mitigating the adverse effects of internet addiction on mental health and overall well-being, ultimately fostering a healthier and more productive future for young individuals and society as a whole.

## Ethics approval statement

The authors complied with APA ethical standards, the study was conducted according to the guidelines of the Declaration of Helsinki, and approved by the Ethics Committee of the Deanship of Scientific Research at Najran University, No (NU/IFC/2/SEHRC/-/19). Participation in the research was voluntary and informed consent was obtained from all participants.

## Consent to participate

Informed consent was obtained from all individual participants included in the study.

## Consent to publish

Not applicable.

## Data Availability

The data is available on request by the corresponding author.
